# Anti-Oxidant and Anti-Melanogenic Properties of Essential Oil from Peel of Pomelo cv. Guan Xi

**DOI:** 10.3390/molecules24020242

**Published:** 2019-01-10

**Authors:** Wanying He, Xiaoyan Li, Ying Peng, Xiaoyan He, Siyi Pan

**Affiliations:** 1 College of Food Science and Technology, Huazhong Agricultural University, Wuhan 430070, Hubei, China; wanyinghe@webmail.hzau.edu.cn (W.H.); lixiaoyanhzau@sina.com (X.L.); pengkangying@webmail.hzau.edu.cn (Y.P.); hexiaoyanhzau@126.com (X.H.); 2 Key Laboratory of Environment Correlative Dietology, Ministry of Education, Huazhong Agricultural University, Wuhan 430070, Hubei, China

**Keywords:** pomelo peel, essential oil, anti-oxidant, anti-melanogenic, B16 melanoma cell

## Abstract

Here, we investigated the anti-oxidant and anti-melanogenic effects of pomelo peel essential oil (PPEO) from pomelo cv. Guan Xi. The volatile chemical composition of PPEO was analyzed with gas chromatography–mass spectrometry (GC/MS). The most abundant component of PPEO was limonene (55.92%), followed by β-myrcene (31.17%), and β-pinene (3.16%). PPEO showed strong anti-oxidant activities against 2,2-diphenyl-2-picryhydrazyl (DPPH), 2,2′-azinobis-(3-ethylbenzthiazoline-6-sulphonate (ABTS) and superoxide anion free radicals. Based on the B16 melanoma cell system, the effects of PPEO on the viability and morphology of B16 cells and the production of melanin were evaluated. The results revealed that PPEO at concentrations below 50 μg/mL could decrease the melanin content without affecting cell viability and morphology. Intracellular tyrosinase (TYR) activity and Western blot analysis showed that PPEO could down-regulate the expression level of TYR in B16 cells and dose-dependently inhibit TYR activity (by a maximum of 64.54%). In conclusion, PPEO has good anti-oxidant and anti-melanogenic activity, and thus can be widely used as a natural antioxidant in the food, pharmaceutical, and cosmetic industries.

## 1. Introduction

Pomelo, a citrus fruit belonging to the genus Rutaceae, is grown and consumed worldwide due to its unique flavor and high nutritional value [1]. In the production of pomelo juice, jam and other products, pomelo peel (PP) is a major by-product accounting for about 50% of the total weight of the fruit. However, most of the PP is disposed of in landfills, resulting in environmental pollution and loss of economic value [2,3]. Pomelo peel contains many natural chemical ingredients, making it a good source of valuable extracts. Compared with the peel of other fruits, PP has a higher concentration of essential oil (EO). Citrus EO is generally considered to be safe with a broad spectrum of biological activities such as anti-inflammatory and anxiolytic effects [4]. Due to its high content of active substances such as terpenes, sesquiterpene, aldehydes, ketones, and esters, pomelo EO has strong aromatic, antioxidant, bacteriostatic, and antiviral properties [4,5], and thus can be used as a functional ingredient and premium fragrance in the food, cosmetic and pharmaceutical industries. Therefore, the high value-added utilization of PP has important research significance and economic prospects.

Oxidative stress can produce reactive oxygen such as superoxide (O_2_), hydrogen peroxide (H_2_O_2_), and hydroxyl radical (HO). These reactive oxygen species can disrupt the balance of normal metabolic activity in the human body and are associated with many chronic diseases such as aging, cancer, atherosclerosis, and inflammation [6]. As a result, research of natural antioxidants has gradually become a hot spot. These antioxidants can remove excess free radicals from the body and relieve conditions caused by excessive free radicals [7,8]. There has been extensive research on the antioxidant activities of plant EOs. Most current research on antioxidants is focused on citrus EOs. For example, the antioxidant properties of 34 kinds of citrus EOs have been tested. The results showed that most of the EOs have good inhibitory effects on 2,2-diphenyl-2-picryhydrazyl (DPPH), which are significantly better than the effects of water-soluble vitamin E [9]. Extract of sweet orange peel have significant inhibitory effects on DPPH, with 50% inhibitory concentrations (IC_50_) of 600 μL/mL [10]. Moreover, the EOs extracted with a cold pressing method also have high antioxidant activity, because cold pressing can better preserve the active ingredients in EOs [11]. However, the above studies are limited to the antioxidant activities of EO mixtures as a whole, while there have been few studies of the antioxidant activities of specific components in EOs.

Melanin is a pigment widely distributed on the surface of skin, hair, retina, and adrenal medulla. It is synthesized from tyrosine under the enzymatic oxidation of tyrosinase (TYR) [12]. However, excessive production and accumulation of melanin can cause pigmentation spots and skin discoloration such as chloasma, freckles, and age spots. Tyrosinase is associated with a variety of diseases and may be a key factor of dopamine neurotoxicity and neurodegeneration associated with Parkinson’s disease [13]. Besides, it is also a key rate-limiting enzyme in the initial reaction of melanin production. At present, the application of EOs as a natural enzyme inhibitor has become a research hotspot. Plant EOs have strong biological activities and great application potentials in biology and medicine [14]. Cinnamon EO and clove EO can inhibit the TYR activity in B16 cells by 37% and 10%, respectively [7]. Lemon EO was observed to have significant inhibitory effects on TYR activity, and its major components were determined to be monoterpenoids and oxindoles [15], which are also the main components of pomelo EO. Hence, it can be speculated that pomelo EO might also have inhibitory effects on TYR activity. Mint leaf EO could reduce the synthesis rate of melanin in B16-F10 cells, and β-caryophyllene (main component) can also decrease melanin production by down-regulating the expression of microphthalmia-associated transcription factor protein (MITF), tyrosinase-related protein-1 (Trp-1), tyrosinase-related protein-2 (Trp-2), and TYR [16]. Aromatic or aliphatic compounds such as anisaldehyde and cuminaldehyde are effective TYR inhibitors [13]. Citrus EOs contain a large amount of fatty aldehyde compounds, indicating their potential inhibitory activity against TYR. However, the current research on pomelo mostly focuses on the storage and preservation of fresh fruit and the extraction of pectin from the peel, while little research attention has been paid to the antioxidant and anti-melanogenic effects of pomelo peel EO (PPEO). Therefore, clarifying the effect of PPEO on melanin synthesis is of great significance for improving the high value-added utilization of PP and expanding its application in the health food, pharmaceutical and cosmetics fields.

In this study, we extracted PPEO from pomelo cv. Guan Xi by a cold pressing method and analyzed its main components using gas chromatography–mass spectrometry (GC/MS). The anti-oxidant activities were examined with 2,2-diphenyl-1-picrylhydrazyl (DPPH); 2,2′-azino-bis(3-ethylbenzothiazoline-6-sulfonic acid) diammonium salt (ABTS), and superoxide anion radical scavenging. Furthermore, based on the B16 melanoma cell system, the effect of PPEO on the viability and morphology of B16 cells and the production of melanin was evaluated. Finally, intracellular TYR activity assay and Western blot analysis were performed to validate the inhibitory effect of PPEO on TYR.

## 2. Results and Discussion

### 2.1. Chemical Composition of Pomelo Peel Essential Oil

**Figure 1:**
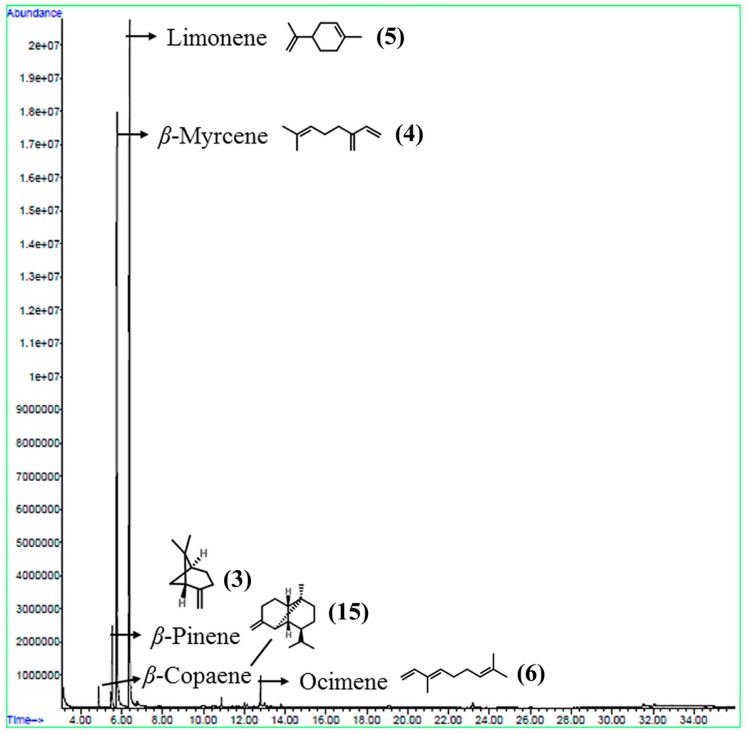
Total ion chromatogram of aroma components from pomelo peel essential oil (PPEO).

The extraction rate of PPEO by the method of cold pressing was 0.82%, which was higher than that in a previous study of citrus essential oils (0.25%) [17]. The obtained PPEO was pale yellow and a clear liquid with a natural aroma. Figure 1 shows the total ion chromatogram obtained from GC/MS analysis of the PPEO 10-fold diluted with absolute ethanol. A total of 21 compounds were detected (Table 1). Among these compounds, 13 were terpenes, which accounted for 94.15% of the total. The highest content of limonene in PPEO was 55.92%, followed by β-myrcene (31.17%), β-pinene (3.16%), ocimene (1.42%), and β-copaene (1.24%) (Table 1). 

### 2.2. Antioxidant Activities of Pomelo Peel Essential Oil 

**Figure 2:**
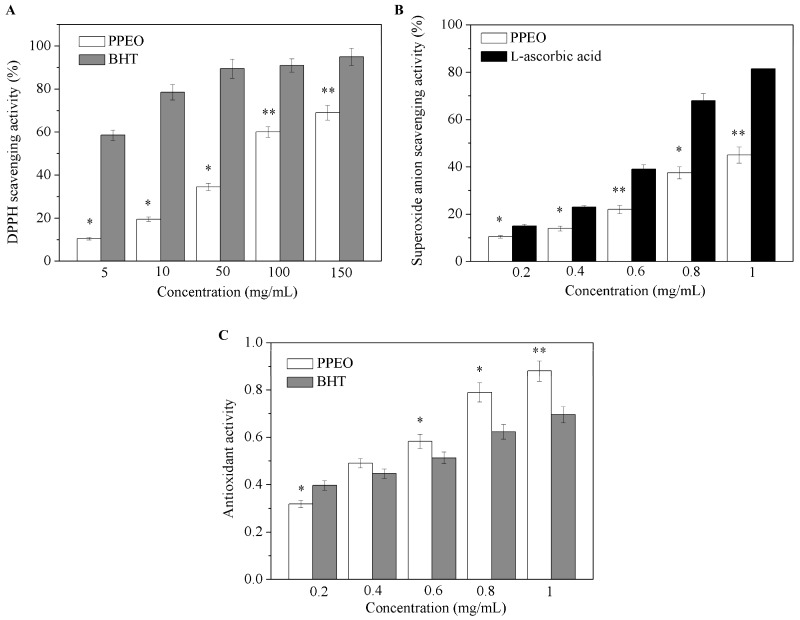
Antioxidant activities of PPEO. (**A**) 1,1-Dipheny-2-Picryhydrazyl (DPPH) radical scavenging assay; (**B**) superoxide anion radical scavenging activity assay; (**C**) 2,2′-azinobis-(3-ethylbenzthiazoline-6-sulphonate) (ABTS) total antioxidant activity. * Indicates samples that are significantly different (*n* = 3; * *p* < 0.05 and ** *p* < 0.01 compared with the positive control group).

Figure 2 shows the DPPH free radical scavenging rate, superoxide anion radical scavenging rate, and total antioxidant activity of PPEO. PPEO exhibited significant effects on the free radical scavenging rate in a concentration dependent manner. At a low concentration (5 mg/mL), PPEO had no significant effect on DPPH free radical scavenging (Figure 2A). However, with increasing concentration, the DPPH free radical scavenging rate of PPEO reached 68.13% at 150 mg/mL, which was statistically significantly different from that of the control group (*p* < 0.01). TheIC_50_ of PPEO was 70.12 mg/mL. The positive control butylated hydroxytoluene (BHT) showed good DPPH free radical scavenging ability at low concentrations. Superoxide anion free radicals can induce lipid peroxidation in the body, thereby accelerating the aging of human skin and even internal organs [18]. As shown in Figure 2B, when the concentration of PPEO was 0.2 mg/mL, the superoxide anion clearance rate was only 11.93% (*p* < 0.05). With increasing PPEO concentration, the clearance rate increased to 44.74% at 1.0 mg/mL (*p* < 0.01). It seems that PPEO could effectively remove the superoxide anion radicals, but with a lower scavenging ability than L-ascorbic acid at the same concentration. Figure 2C shows that the total antioxidant activity of PPEO was slightly lower than that of BHT at the concentrations lower than 0.4 mg/mL. However, as the concentration increased, the antioxidant activity of PPEO exceeded that of BHT. At the concentration of 1.0 mg/mL, the total antioxidant activity of PPEO was 20.35% higher than that of BHT. These results indicated that low-concentration PPEO has no obvious antioxidant effect while high-concentration PPEO has good antioxidant effect. Butylated hydroxytoluene is an industrially synthesized antioxidant that facilitates fast oxidation resistance at low concentrations, while PPEO is a mixture of various compounds with antioxidant activities, which may have complex interactions with each other [19].

### 2.3. Anti-Melanogenic Effects of Pomelo Peel Essential Oil

#### 2.3.1. Effect of Pomelo Peel Essential Oil on Cell Viability

**Figure 3:**
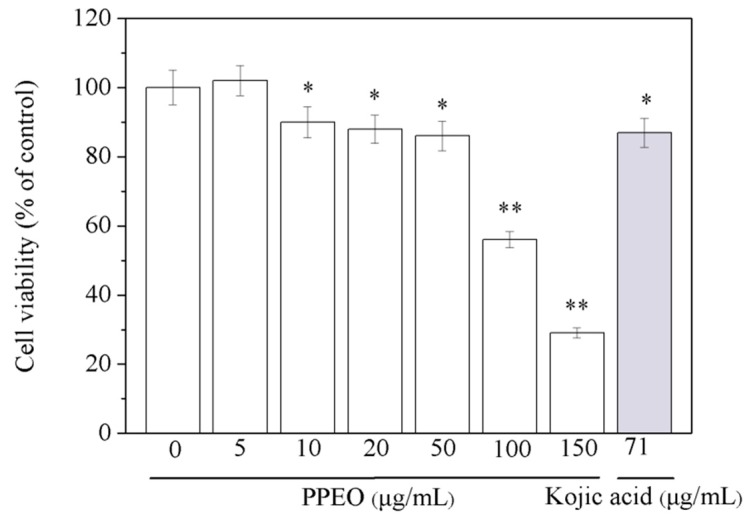
Effect of PPEO on B16 melanoma cell viability. * Indicates samples that are significantly different (*n* = 3; * *p* < 0.05 and ** *p* < 0.01 compared with the blank control group).

B16 melanoma cells were treated with different concentrations of PPEO for 24 h, and the viability of each group was detected by MTT assay (Figure 3). The survival rate of B16 cells treated with a low concentration of PPEO (5 μg/mL) was higher than 100%, indicating that low concentration of PPEO may facilitate the proliferation of B16 cells, which might be related to the active volatile components in PPEO [20]. The cell viability decreased significantly along with increasing PPEO concentration. When the concentration of PPEO was 150 μg/mL, the cell survival rate significantly decreased to 29.35% (*p* < 0.01). The results show that at concentrations lower than 50 μg/mL, PPEO did not affect cell viability. However, high concentrations of PPEO (>50 μg/mL) significantly inhibited cell viability.

#### 2.3.2. Effect of Pomelo Peel Essential Oil on Cell Morphology

**Figure 4:**
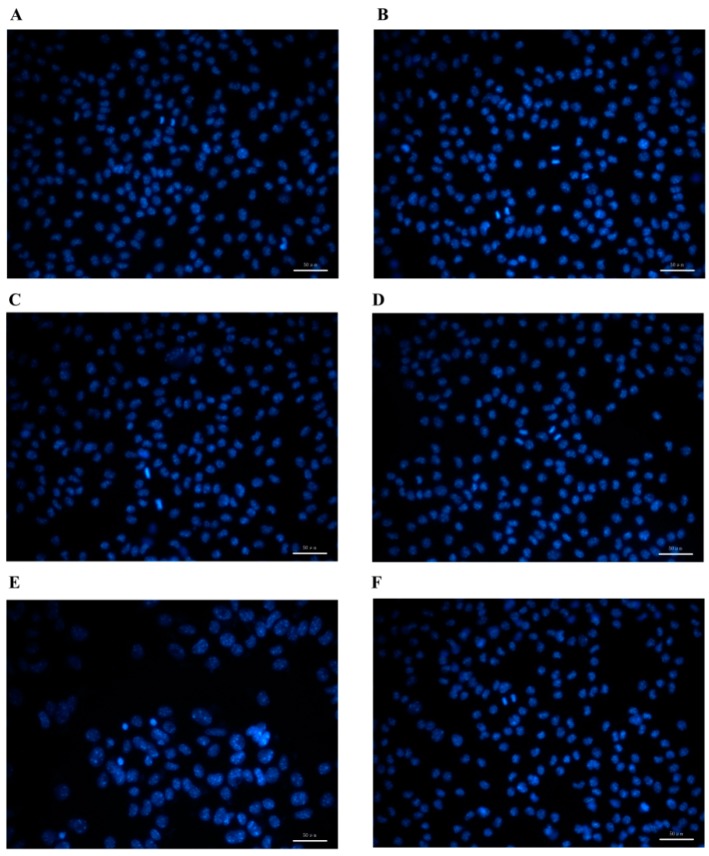
Optical microscopic morphology of B16 cells. PPEO at concentrations of 0, 10, 20, 50, 100 μg/mL were for (**A**–**E**), respectively, and kojic acid at a concentration of 71 μg/mL was for (**F**). Scale bar: 50 μm

B16 cells are adherent and mostly fusiform cells with dendrites and relatively more divisions. They are tightly connected monolayers with high transparency (Figure 4). The cells in the control group showed uniform fluorescence, clear cell boundaries, and normal dendritic morphology. When the concentration of PPEO was 10–50 μg/mL, the number of cell deaths was small, the cell boundary was clear, and the fluorescence was relatively uniform. However, at a concentration of 100 μg/mL, the number of cell deaths increased and the boundaries between cells became blurred, accompanied by the appearance of obvious fluorescent spots. At the same time, cells were dispersed and the dendrites were reduced. In the high-concentration PPEO group (100 μg/mL), the cells showed enhanced fluorescence, and were swollen and separated from each other, presenting a typical state of apoptosis in the cells. 

#### 2.3.3. Inhibition of Pomelo Peel Essential Oil on Intracellular Tyrosinase Activity and Melanin Content 

**Figure 5:**
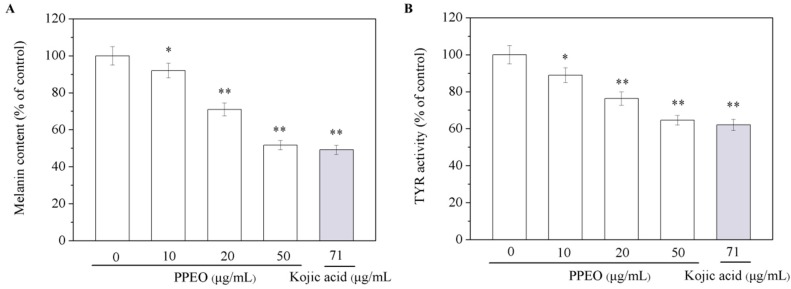
Effect of PPEO on melanin content (**A**) and tyrosinase activity (**B**) in B16 cells. * Indicates samples that are significantly different (*n* = 3; * *p* < 0.05 and ** *p* < 0.01 compared with the blank control group).

To determine the anti-melanogenic activity of PPEO, we evaluated its effect on TYR activity and melanin content in B16 melanoma cells. The B16 melanoma cells were treated with various concentrations of PPEO and then co-cultured for 72 h. As shown in Figure 5, PPEO dose-dependently inhibited TYR activity and melanin content. At a concentration of 50 μg/mL, melanin synthesis and TYR activity were inhibited by 48.28% and 64.54%, respectively, and the IC_50_ of melanin synthesis in inhibition was 67.64 μg/mL. Kojic acid, the positive control, inhibited TYR activity by 62.09%, which is similar to the inhibitory effect of PPEO at 50 μg/mL (Figure 5B). 

#### 2.3.4. Effect of Pomelo Peel Essential Oil on Tyrosinase Expression in B16 Cells 

**Figure 6:**
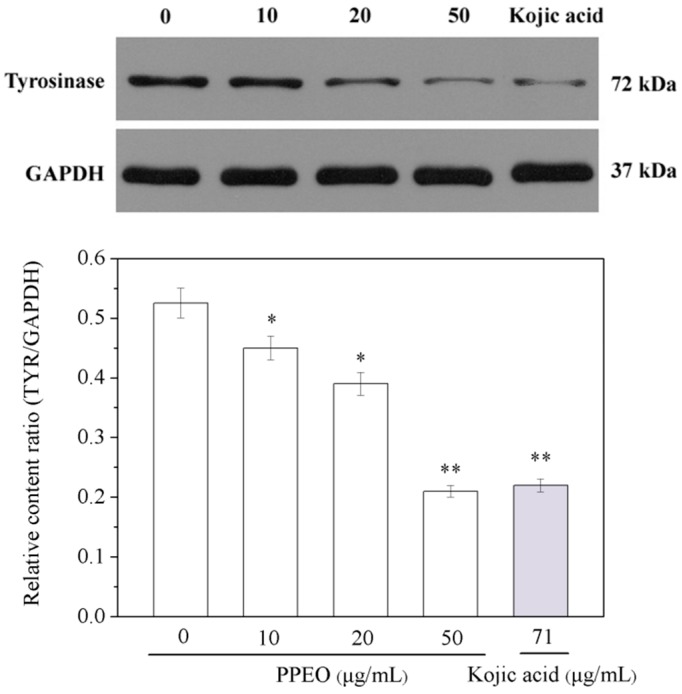
Effect of PPEO on tyrosinase expression in B16 cells. * Indicates samples that are significantly different (*n* = 3; * *p* < 0.05 and ** *p* < 0.01 compared with the blank control group). GADPH: glyceraldehyde-3-phosphate dehydrogenase.

The expression of TYR protein was detected by Western blotting, and the intensity of protein expression was determined by the ratio of the target band to the internal reference band (Figure 6). PPEO down-regulated the expression level of TYR in B16 cells in a concentration-dependent manner. Compared with that in the blank control group, the TYR expression gradually decreased with increasing PPEO concentration. When the PPEO concentration was 50 μg/mL, the TYR expression was 60.38% lower than that of the blank group and was close to that of the positive group (kojic acid). This result is consistent with the tyrosinase enzyme linked immunosorbent assays (ELISA) test results. 

### 2.4. Discussion

Previous studies have shown that the characteristic aroma of pomelo is mainly attributable to a variety of compounds [2]. In this study, we first extracted PPEO from pomelo cv. Guan Xi and analyzed its main components using GC/MS. Our study demonstrated that the fresh and natural fruit aromas were mainly due to the presence of aldehydes and terpenoids in PPEO. The safety of natural products used in health foods, drug, and cosmetic ingredients is a major concern. Several studies have explored the use of extracts from pulp of guava [21], waste of citrus [6], earthworm [18], oil of *Aquilaria crassna* [8], and oil from *Alpinia zerumbet* [22]. We first determined the antioxidant activities of PPEO. Previous studies have shown that plant EOs have universal antioxidant activities [14]. Meanwhile, it has been reported that terpenes such as limonene, β-myrcene, β-pinene, ocimene, β-copaene, and citral showed anti-oxidant activities [21,23,24]. The strong anti-oxidant activity of PPEOs may be attributable to these components. In our experiment, PPEO was extracted using a cold pressing method under normal temperature conditions, which could better preserve the active components with antioxidant function, resulting in a high total antioxidant capacity. It is known that ultra violet (UV) radiation induces free radical formation in the skin, which is linked directly to the onset of skin photodamage and biological damage. Thus, our results suggest that PPEO may be a useful anti-oxidant source and have the potential to prevent UV-induced damage.

Next, we highlighted the anti-melanogenic effects of PPEO through cell viability, cell morphology, intracellular melanin content, intracellular TYR activity and expression, and compared these results with those of the positive control (kojic acid). Previous studies have shown that the action mechanism of terpenoids in cells is related to the destruction of lipophilic compounds in biofilms [25,26]. Due to the high hydrophobicity of terpenoids, their toxic effects lead to swelling and enhanced fluidity and permeability of the cell membranes [27]. Therefore, terpenoids might cause death of cells at high concentrations of PPEO. However, when the concentration of PPEO was below 50 μg/mL, the state of the cell was normal. On considering this possibility, concentrations below 50 μg/mL of PPEO were used to evaluate its effects on melanin content and intracellular TYR activity.

To our knowledge, TYR catalyzes the first two steps of mammalian melanogenesis [18], namely the hydroxylation of monophenol to *o*-diphenol and the oxidation of diphenol to *o*-quinones, both of which use molecular oxygen, followed by a series of nonenzymatic steps to finally result in the formation of melanin [12]. Therefore, inhibition of TYR activity may help to avoid abnormal melanin pigmentation in skin. The results of this study preliminarily demonstrated the good antioxidant performance of PPEO. In the co-culture of PPEO and B16 cells, PPEO acted as an antioxidant to inhibit the catalytic reaction of TYR and block the synthesis pathway of melanin, resulting in a decrease in melanin production. Meanwhile, the effect of PPEO on the dendritic morphology of cells might also destroy the normal physiological functions of cells, which in turn affects the formation of melanin in cells. The above results demonstrate that PPEO can decrease the melanin content without affecting the cell viability.

In addition, previous study showed that citral, myrcene, (2E)-alkenal, and terpinolene were popular tyrosinase inhibitors [13]. In another study, citral and myrcene were found to have significant inhibitory effects on TYR, and trans-citral has better inhibitory effect than *cis*-citral. In plant EOs, the content of *trans*-citral is higher than that of *cis*-citral. Meanwhile, citral and myrcene are the main active substances that inhibit TYR in EOs [28]. Therefore, it is most likely that the inhibition of TYR expression by the components (limonene, β-myrcene, β-pinene, ocimene, β-copaene) in PPEO is a synergistic effect, and the main active components could be partly attributed to citral and myrcene.

## 3. Materials and Methods

### 3.1. Materials

Fully ripe fruits of pomelo cv. Guan Xi were harvested from Fujian province of China. Reagents 2,2-diphenyl-1-picrylhydrazyl (DPPH), dimethyl sulfoxide (DMSO), Hoechst 33258 staining solution, fetal bovine serum (FBS), *n*-paraffins (C7–C30), and tyrosinase enzyme linked immunosorbent assays (ELISA) kit were purchased from Sigma-Aldrich Co. (St. Louis, MO, USA). Dulbecco’s Modified Eagle Medium (DMEM) was from Gibco Chemical Co. (Grand Island, NY, USA). Penicillin-streptomycin double antibody, trypsin, and methyl thiazolyl tetrazolium (MTT) were purchased from Genivew Co. (El Monte, CA, USA). Cell Lysates, tert-butyl hydroxytoluene (BHT), kojic acid, and protein quantification test kit were purchased from Shanghai Yuye biotechnology Co., Ltd. (Shanghai, China). RPMI 1640 medium was purchased from Hyclone Co. (Logan, UT, USA). ECL chemiluminescence detection kit and sodium dodecyl sulfate polyacrylamide gels (SDS-PAGE) Gel Preparation Kit were purchased from Aspentech Co. (Bedford, MA, USA). B16 melanoma cells were purchased from Shanghai Tongpai Biotechnology Co., Ltd (Shanghai, China). All other analytical grade chemicals were bought from Sinopharm chemical reagent Co., Ltd (Shanghai, China).

### 3.2. Extraction of Essential Oil 

Fresh pomelo was picked and the white peel from the fresh pomelo peel was removed, and then the exocarp chopped in 1.5 cm × 0.5 cm × 0.2 cm sized pieces. Pomelo peel (100 g) was crushed and pressed twice with a cold hydraulic press (Model 6YL-190; Changbai Mountain Technology Limited company, Changchun, China) [28] and then the peel slurry was collected. After filtering through a steel sieve (0.15 mm), saturated sodium chloride (NaCl) solution was added to the sample for the extraction of PPEO for 3 h. Then, the sample was centrifuged at 10,000× *g* for 30 min at 4 °C. Supernatants were stored in separate amber bottles at −20 °C until use. The extracted PPEO was weighed to determine the extraction yield as follows: extraction yield (%) = [(weight of extracted oil)/(weight of pomelo peel)] × 100%.

### 3.3. Gas Chromatography-Mass Spectrometry Analysis

The extracted PPEO was filtered through a 0.45 μm microporous organic membrane. Volatile compounds were analyzed using an Agilent 7890A GC coupled to an Agilent 5975C mass spectrometer (Palo Alto, CA, USA). The components of PPEO were identified using HP-5Ms phenylmethylsiloxane capillary column (30 m × 0.25 mm i.d., 0.25 μm; Agilent Technologies, J & W Scientific Products, Folsom, CA, USA) [24]. The helium was used as a carrier gas with a flow rate of 1 mL/min. Injector temperature was 250 °C. The split ratio was 10:1. The temperature program was 45 °C (hold for 1 min), increase at 10 °C/min to 165 °C (hold for 2 min), increase at 1.5 °C/min to 180 °C (hold for 2 min), and then increase at 10 °C/min to 250 °C (hold for 2 min). The temperature of both injector and detector was set at 250 °C. Mass spectra were scanned from *m/z* 35–350 amu. The electron impact ionization energy was 70 eV. Identification of compounds detected by GC/MS analysis was performed by comparing mass spectra and retention indices (RIs) with published data obtained under similar conditions, as well as by comparing their mass spectra with the MS library of Wiley 7.0 and Nist 05 [29]. A mixture of *n*-paraffins (C7–C30) as standards was used for calculating RIs. Samples were analyzed and identified using an available Retention Time Locked (RTL) database with Deconvolution Reporting Software (DRS) and a database of 926 DRS compounds.

### 3.4. Antioxidant Activities

#### 3.4.1. DPPH Radical Scavenging Assay

The DPPH radical scavenging assay was performed according to Boskou et al. [23]. The sample PPEO was prepared with different concentrations with absolute ethanol. A mixture of 50 μL sample and 150 μL 0.1 mmol/L DPPH free radical ethanol solution was taken and placed in a 96-well plate. After vigorous shaking, the mixture was incubated at room temperature in the dark for 30 min. The absorbance at 517 nm was measured. Pomelo peel essential oil was replaced with absolute ethanol to serve as the blank control, and BHT was used as positive control.

#### 3.4.2. Superoxide Anion Radical Scavenging Activity Assay

The superoxide anion radical scavenging activity was measured as described by Zhang et al. [30]. The reaction mixture consisted of 4.5 mL 50 mM Tris-HCl buffer (pH 8.2) and 1 mL sample solution at different concentrations. The mixed solution was pre-incubated at 25 °C for 10 min, and then initiated by the addition of 0.45 mL 2.5 mM pyrogallol. After vigorous shaking for 5 min, the reaction was terminated by the addition of 8 mol/L HCl. The absorbance was read at 517 nm.

#### 3.4.3. ABTS Radical Scavenging Assay

The ABTS radical scavenging assay was conducted following the method previously described by Re et al. [31]. Diluted radical solution was prepared by mixing 7 mM ABTS and 2 mM K_2_S_2_O_8_ in equal amounts, followed by reaction in the dark overnight at room temperature. The samples were prepared in different concentrations with ultrapure water. Aliquots of 10 μL of samples were mixed with 200 μL of the diluted radical solution in 96-well plate and the absorbance was measured at 734 nm after 5 min using an M200 pro enzyme-labeled instrument (Tecan, Ltd., Männedorf, Switzerland). BHT was used as the positive control.

### 3.5. Cell Culture and Treatment

The murine metastatic melanoma cell line B16 was cultured in sterile cell culture flasks with RPMI 1640 medium supplemented with 100 U/mL penicillin, 100 U/mL streptomycin, and 10% heat inactivated FBS at 37 °C in a humidified incubator containing 5% CO_2_. Cells in logarithmic growth phase were selected for subsequent experiments [32]. The extracted PPEO was dissolved in Tween 80 and filtered through a 0.45 μm microporous organic membrane. The PPEO was diluted to different concentrations and then was added to the medium.

### 3.6. MTT Assay for Cell Viability

Cell viability was evaluated by 3-[4, 5-dimethylthiazol-2-yl]-2, 5-diphenyl tetrazolium bromide (MTT) assay according to the method of Satooka et al. [33]. The cell density was 7 × 10^4^ cells/mL and the cells were seeded on 96-well cell culture plates at 100 μL per well. After 24 h of culture, the original culture solution was aspirated. Cells were exposed to various concentrations of PPEO or kojic acid (71 μg/mL), with 6 replicates for each concentration (500 μmol/L of kojic acid is equal to 71 μg/mL). After culture for another 24 h, 100 μL of 0.5 mg/mL MTT was added to each well, followed by inoculation for 4 h at 37 °C. The liquid was carefully aspirated from the well, and then 150 μL of DMSO was added to each well. After 10 min of shaking, the absorbance was measured at 490 nm using an M200 pro enzyme-labeled instrument (Tecan, Ltd.).

### 3.7. Immunofluorescence Analysis and Hoechst Staining

A sterilized coverslip was placed into each hole of the six-hole plate on an ultra-clean workbench. The cell suspension was added to each coverslip and placed in an incubator with a CO_2_ concentration of 5% at 37 °C until cell fixation (2 h). After the addition of 2 mL culture medium, the culture was continued for about 6 h. The medium was decanted and the cells were washed for 5 min with PBS for 3 times. The cells were fixed with 4% paraformaldehyde for 30 min, and then paraformaldehyde was removed by PBS buffer washing. After the addition of appropriate amounts of Hoechst stain, the coverslips were incubated at room temperature for 15 min in the dark. The coverslips were then rinsed 3 times with PBS for 5 min each time, and the side with the cells was observed under a confocal laser scanning microscope [34].

### 3.8. Determination of Melanin Content

Melanin content was determined as described by Huang et al. [35]. B16 cells were plated at a density of 7 × 10^4^ cells/well in a 6-well plate. The experimental group was added with 0.2 μmol/L α-MSH (melanocyte-stimulating hormone) to construct a cell model with high melanin expression. After 12 h of culture, cells were exposed to various concentrations of PPEO (10–100 μg/mL). Kojic acid at a concentration of 71 μg/mL was used as a positive control. After 48 h of culture, the supernatant was discarded and the cells were washed 3 times with PBS buffer. After the addition of 200 μmol/L NaOH solution (containing 10% DMSO) to each well, the cells were fully lysed at 80 °C for 1 h, and the absorbance was measured at 492 nm. The amount of protein was measured by Micro BCA protein assay kit (Shanghai Yuye biotechnology Co., Ltd, Shanghai, China). The melanin content was calculated by normalization to the total cellular protein (1 g of melanin/mg of protein) and reported as a percentage of the control.

### 3.9. Intracellular Tyrosinase Activity

B16 cells were plated at a density of 7 × 10^4^ cells/well in a 6-well plate. After 24 h of culture, cells were exposed to various concentrations of PPEO (10–100 μg/mL) or kojic acid (71 μg/mL), and incubated for additional 48 h. The cells were then washed with ice-cold phosphate buffer. The plates were frozen at −80 °C for 30 min. After thawing and mixing, the tyrosinase activity was measured by ELISA kit [36].

### 3.10. Protein Extraction and Western Blot Analysis

Total protein was extracted from cells lysed by RIPA Lysis buffer with 1% phenylmethylsulfonyl fluoride (PMSF). The amount of protein was measured by Micro BCA protein assay kit (Shanghai Yuye biotechnology Co., Ltd.). The samples were loaded on 10% sodium dodecyl sulfate polyacrylamide gels (SDS-PAGE) and transferred onto polyvinylidene difluoride (PVDF) membranes. The membranes were then placed in blocking solution and blocked at room temperature for 1 h. The membranes were incubated overnight at 4 °C with appropriate concentrations of specific antibodies, including rabbit monoclonal antibodies glyceraldehyde-3-phosphate dehydrogenase (GAPDH, 1:10000 dilution) and rabbit monoclonal antibodies TYR (1:1000 dilution). After five or six times of washing, the blots were then incubated with secondary antibody (HRP-Goat anti Rabbit). BandScan was used to analyze the integrated density of bands.

### 3.11. Statistical Analysis

All the experiments were performed with freshly prepared samples in triplicate. The results were expressed as means ± standard deviation (SD) and analyzed by one-way analysis of variance (ANOVA) test using SPSS 19.0 (IBM Corporation, Armonk, NY, USA). Differences were considered as statistically significant at the level of *p* < 0.05.

## 4. Conclusions

Our study is the first to extract essential oil from the peel of pomelo cv. Guan Xi by a cold pressing method and analyze its main components of limonene, β-myrcene, β-pinene, ocimene, and β-copaene. Our results reveal that PPEO has strong antioxidant activities against DPPH, ABTS, and superoxide anion radicals and the main active components responsible for the effect are terpenes. Besides, the effects of PPEO on the viability of B16 cells and the production of melanin were evaluated based on the B16 melanoma cell system. The results indicate that PPEO down-regulates the expression level of TYR in B16 cells, which inhibits the catalytic reaction of TYR and blocks the synthesis pathway of melanin; and it further reduces melanin production without affecting the cell viability. This study provides data support for expanding the potential application of essential oil from pomelo peel as a natural antioxidant in the food, pharmaceutical and cosmetic industries. Further research in vivo is needed to fully evaluate the potential anti-melanogenic effect of PPEO.

## Figures and Tables

**Table 1:** Chemical components of essential oils.

No.	Compound	Retention Index	Molecular Formula	Peak Area (%)
1	α-Pinene	951	C_10_H_16_	0.15
2	Artemisia triene	966	C_10_H_6_	0.05
3	β-Pinene	994	C_10_H_16_	3.16
4	β-Myrcene	1009	C_10_H_16_	31.17
5	Limonene	1050	C_10_H_16_	55.92
6	Ocimene	1073	C_10_H_16_	1.42
7	Propionamide	1149	C_9_H_11_NO	0.40
8	Metaraminol	1295	C_9_H_13_NO_2_	0.21
9	Citral	1348	C_10_H_16_O	0.73
10	4-Carene	1366	C_10_H_16_	0.38
11	Norephedrine	1382	C_9_H_13_NO	0.04
12	Caryophyllene	1418	C_15_H_24_	0.13
13	Cubebene	1463	C_15_H_24_	0.15
14	Cathinone	1498	C_9_H_11_NO	0.05
15	β-Copaene	1516	C_15_H_24_	1.24
16	Bicyclogermacrene	1523	C_15_H_24_	0.24
17	γ-Elemene	1596	C_15_H_24_	0.10
18	2,6,11,15-Tetramethyl-hexadeca-2,6,8,10,14-pentaene	1979	C_20_H_30_O_2_	0.50
19	β-Farnesene	2014	C_15_H_24_	0.18
20	7-Methoxy-6-(3-methyl-2-oxobutyl)-2*H*-1-benzopyran-2-one	2302	C_16_H_16_O_4_	0.61
21	2-(Methylamino)-1-phenylethanol	2329	C_9_H_13_NO	0.04
	Total			96.87

## Supplementary Materials

Supplementary File 1
