# Supplementary material for: Anti-Oxidant and Anti-Melanogenic Properties of Essential Oil from Peel of Pomelo cv. Guan Xi
Source: Molecules. 2019 Jan 10;24(2):242. doi: 10.3390/molecules24020242 (PMC6359654; doi:10.3390/molecules24020242)
Supplement: Supplementary File 1 [file molecules-24-00242-s001.zip › n-paraffins (C7¿CC30) data by GC¿CMS analysis/tmplibrp.pdf]

谱库检索报告

数据路径 : C:\msdchem\1\data\20181218\
数据文件 : HN-STA-1.D
采集 : 18 Dec 2018 9:36
操作者 :
样品 :
其他 :
ALS 样品瓶: 1 样品乘积因子: 1

检索库: C:\Database\NIST14.L 最小匹配度: 0

未知谱图: 顶点
积分事件: 化学工作站积分器 - events.e

峰#	R.T.	面积%	谱库/ID	参比#	CAS#	匹配度
1	4.118	2.62	C:\Database\NIST14.L			
			Nonane	12938	000111-84-2	94
			Nonane	12937	000111-84-2	87
			Nonane	12939	000111-84-2	68
2	5.621	2.87	C:\Database\NIST14.L			
			Decane	19649	000124-18-5	95
			Decane	19650	000124-18-5	91
			Decane	19648	000124-18-5	87
3	7.147	3.16	C:\Database\NIST14.L			
			Undecane	29357	001120-21-4	96
			Undecane	29356	001120-21-4	96
			Undecane	29355	001120-21-4	91
4	8.623	3.38	C:\Database\NIST14.L			
			Dodecane	39973	000112-40-3	97
			Dodecane	39974	000112-40-3	96
			Dodecane	39972	000112-40-3	96
5	10.022	3.58	C:\Database\NIST14.L			
			Tridecane	51394	000629-50-5	98
			Tridecane	51393	000629-50-5	97
			Tridecane	51395	000629-50-5	97
6	11.345	3.72	C:\Database\NIST14.L			
			Tetradecane	63625	000629-59-4	98
			Tetradecane	63622	000629-59-4	98
			Tetradecane	63623	000629-59-4	96
7	12.207	0.05	C:\Database\NIST14.L			
			2,6-Dihydroxyacetophenone, 2TMS derivative	155019	1000352-81-3	38
			5-Methylsalicylic acid, 2TMS derivative	155018	1000153-59-4	32
			1-Pentene, 1,3-diphenyl-1-(trimethylsilyloxy)-	169273	138983-00-3	32
8	12.597	3.45	C:\Database\NIST14.L			
			Pentadecane	76609	000629-62-9	97
			Pentadecane	76606	000629-62-9	95
			Pentadecane	76608	000629-62-9	94
9	13.868	4.02	C:\Database\NIST14.L			

			Hexadecane	89842	000544-76-3	99
			Hexadecane	89843	000544-76-3	95
			Hexadecane	89840	000544-76-3	95
10	14.820	0.50	C:\Database\NIST14.L			
			Malonic acid, bis(2-trimethylsilyl ethyl ester	162858	090744-45-9	14
			3-Isopropoxy-1,1,1,5,5,5-hexamethyl-3-(trimethylsiloxy)trisiloxane	208113	072182-11-7	12
			Boric acid, 3TMS derivative	138625	004325-85-3	10
11	15.547	4.07	C:\Database\NIST14.L			
			Heptadecane	102598	000629-78-7	98
			Heptadecane	102599	000629-78-7	98
			Heptadecane	102600	000629-78-7	97
12	17.744	4.14	C:\Database\NIST14.L			
			Octadecane	115547	000593-45-3	98
			Octadecane	115544	000593-45-3	93
			Hexadecane	89844	000544-76-3	91
13	18.464	0.19	C:\Database\NIST14.L			
			Oxalic acid, 2TMS derivative	97005	018294-04-7	22
			2-Pentenoic acid, 2-[(trimethylsilyl)oxy]-, trimethylsilyl ester	120478	055045-17-5	22
			Pentasiloxane, 1,1,3,3,5,5,7,7,9,9-decamethyl-	209739	000995-83-5	16
14	20.525	4.24	C:\Database\NIST14.L			
			Nonadecane	128835	000629-92-5	98
			Nonadecane	128834	000629-92-5	95
			Heptadecane	102599	000629-78-7	91
15	23.888	4.62	C:\Database\NIST14.L			
			Eicosane	142238	000112-95-8	99
			Eicosane	142239	000112-95-8	98
			Heptadecane	102598	000629-78-7	96
16	27.843	4.25	C:\Database\NIST14.L			
			Heneicosane	155888	000629-94-7	98
			Heneicosane	155886	000629-94-7	96
			Octadecane	115546	000593-45-3	95
17	29.567	1.17	C:\Database\NIST14.L			
			cis-Vaccenic acid	142073	000506-17-2	99
			Oleic Acid	142072	000112-80-1	99
			9-Octadecenoic acid	142074	002027-47-6	98
18	30.321	4.63	C:\Database\NIST14.L			
			Docosane	169409	000629-97-0	98
			Hexadecane, 2,6,10,14-tetramethyl-	142261	000638-36-8	98
			Docosane	169408	000629-97-0	94
19	31.749	0.15	C:\Database\NIST14.L			
			3-Isopropoxy-1,1,1,5,5,5-hexamethyl-3-(trimethylsiloxy)trisiloxane	208113	072182-11-7	30
			Benzyloxyamine, 2TMS derivative	127249	079208-41-6	20
			3-Phenylpyrrolidine	22873	062624-46-8	15
20	31.936	4.64	C:\Database\NIST14.L			
			Tricosane	182654	000638-67-5	98

			Hexadecane, 2,6,10,14-tetramethyl-	142261	000638-36-8	98
			Octadecane	115547	000593-45-3	96
21	33.196	4.61	C:\Database\NIST14.L			
			Tetracosane	195673	000646-31-1	99
			Tetracosane	195670	000646-31-1	99
			Tetracosane	195672	000646-31-1	99
22	33.402	0.25	C:\Database\NIST14.L			
			9-Octadecenamide, (Z)-	141028	000301-02-0	94
			9-Octadecenamide, (Z)-	141027	000301-02-0	86
			Cyclopropaneoctanal, 2-octyl-	140252	056196-06-6	76
23	33.529	0.32	C:\Database\NIST14.L			
			3-Isopropoxy-1,1,1,5,5,5-hexamethyl-3-(trimethylsiloxy)trisiloxane	208113	072182-11-7	38
			2-Amino-4-(4-nitrophenyl)thiazole	84656	002104-09-8	15
			Hexahydropyridine, 1-methyl-4-[4-hydroxy-3-methoxyphenyl]-	84391	094427-44-8	15
24	33.691	0.37	C:\Database\NIST14.L			
			6-Octadecenoic acid	142075	1000336-66-8	91
			Oleic Acid	142071	000112-80-1	55
			cis-11-Hexadecenal	100562	053939-28-9	50
25	34.110	0.14	C:\Database\NIST14.L			
			Benzo[3,4]cyclobuta[1,2]cyclooctene, 4b,5,6,7,8,9,10,10a-octahydro-	53117	056666-89-8	38
			Indole, 3-methyl-	14275	000083-34-1	30
			1H-Indole, 6-methyl-	14293	003420-02-8	30
26	34.268	4.53	C:\Database\NIST14.L			
			Octadecane	115547	000593-45-3	96
			Heptadecane	102599	000629-78-7	95
			Heptadecane, 3-methyl-	115552	006418-44-6	95
27	35.063	0.15	C:\Database\NIST14.L			
			Piperidine, 1-(5-trifluoromethyl-2-pyridyl)-4-(1H-pyrrol-1-yl)-	154311	1000268-74-7	25
			Ambrox	98677	100679-85-4	25
			Trimethylsilyl-di(timethylsiloxy)-silane	140353	139347-50-5	25
28	35.411	4.65	C:\Database\NIST14.L			
			Octadecane	115547	000593-45-3	96
			Heptadecane	102599	000629-78-7	95
			Octacosane	235614	000630-02-4	94
29	35.685	0.52	C:\Database\NIST14.L			
			phenol, 2,6-dichloro-3-methyl-4-nitro-	84566	1000400-81-4	68
			1,3,4-Oxadiazole-2-acetic acid, 5-(1,3-benzodioxol-5-yl)-	109130	1000338-39-3	22
			L-Rhamnose, (R,R,S,S)-, 4TMS derivative	256958	108392-01-4	22
30	36.210	0.20	C:\Database\NIST14.L			
			3-Phenyl-4,5-dimethyl-2,1-oxaborolane	66780	1000062-26-1	43
			2(3H)-Thiazolethione, 4-methyl-	14107	005685-06-3	43
			1H-Indole, 1-methyl-	14299	000603-76-9	43

31	36.630	4.87	C:\Database\NIST14.L			
			Octadecane	115547	000593-45-3	96
			Heptadecane	102599	000629-78-7	95
			Nonadecane, 9-methyl-	142247	013287-24-6	95
32	36.757	0.32	C:\Database\NIST14.L			
			9H-carbazole, 3-ethenyl-9-ethyl-	84521	1000400-80-8	35
			benzenamine, N-[bis(2,4,6-trimethylphenyl)boryl]-	198391	1000398-20-2	30
			4H-Pyrido[1,2-a]pyrimidine-3-carboxamide, 1,6,7,8-tetrahydro-1,6-dimethyl-4-oxo-	84145	064399-29-7	25
33	37.019	0.09	C:\Database\NIST14.L			
			5-Hexenoic acid, 6-[p-chlorophenyl]-2,4-dioxo-, ethyl ester	139543	076781-59-4	51
			Benzo[h]quinoline, 2,4-dimethyl-	71668	000605-67-4	48
			2-(n-Propyl)oxybenzylidene acetophenone	126759	1000395-75-6	42
34	37.503	6.25	C:\Database\NIST14.L			
			Tetracosane	195673	000646-31-1	97
			Heptadecane	102599	000629-78-7	95
			Octadecane	115547	000593-45-3	95
35	37.642	0.37	C:\Database\NIST14.L			
			Pyrrolidine, 1-(9-borabicyclo[3.3.1]non-9-yl)-	56917	022516-41-2	35
			1,3,4-Oxadiazole-2-acetic acid, 5-(1,3-benzodioxol-5-yl)-	109130	1000338-39-3	25
			2-Fluoro-6-trifluoromethylbenzamide, N-pentyl-	136981	1000358-11-1	25
36	37.863	0.39	C:\Database\NIST14.L			
			Phthalic acid, 2TMS derivative	168551	002078-22-0	47
			3-Phenylpyrrolidine	22873	062624-46-8	15
			2-Carboxycinnamic acid	57260	000612-40-8	15
37	38.182	5.25	C:\Database\NIST14.L			
			Nonadecane, 9-methyl-	142250	013287-24-6	95
			Octacosane	235614	000630-02-4	95
			Dotriacontane, 1-iodo-	272203	1000406-32-4	95
38	38.669	0.84	C:\Database\NIST14.L			
			2-Ethylacridine	71643	055751-83-2	47
			2,4-Cyclohexadien-1-one, 3,5-bis(1,1-dimethylethyl)-4-hydroxy-	85540	054965-43-4	47
			Benzo[h]quinoline, 2,4-dimethyl-	71668	000605-67-4	38
39	38.759	4.84	C:\Database\NIST14.L			
			Tricosane	182654	000638-67-5	97
			Tetracosane	195673	000646-31-1	97
			Heptadecane	102599	000629-78-7	96
40	38.931	0.29	C:\Database\NIST14.L			
			2-Ethylacridine	71643	055751-83-2	60
			Thymol, TBDMS derivative	124822	330455-64-6	55
			acetic acid, 2-[bis(methylthio)methylene]-1-phenylhydrazide	114619	1000401-44-4	46

41	39.362	0.75	C:\Database\NIST14.L			
			Thymol, TBDMS derivative	124822	330455-64-6	46
			N-Methyl-1-adamantaneacetamide	71587	031897-93-5	38
			Benzo[h]quinoline, 2,4-dimethyl-	71668	000605-67-4	38
42	39.475	0.36	C:\Database\NIST14.L			
			4-Dehydroxy-N-(4,5-methylenedioxy-2-nitrobenzylidene)tyramine	157264	1000111-66-9	43
			2-(n-Propyl)oxybenzylidene acetophenone	126759	1000395-75-6	38
			2-(Acetoxymethyl)-3-(methoxycarbonyl)biphenylene	141906	093103-70-9	38
43	39.625	0.16	C:\Database\NIST14.L			
			2-Ethylacridine	71643	055751-83-2	59
			1,2-Benzisothiazol-3-amine, TBDMS derivative	124363	1000332-57-2	50
			1-(4-Chlorophenoxy)-1-(1H-imidazol-1-yl)-3,3-dimethylbutan-2-one	151396	1000408-59-9	47
